# CASCADE: Dataset of extant coccolithophore size, carbon content and global distribution

**DOI:** 10.1038/s41597-024-03724-z

**Published:** 2024-08-24

**Authors:** Joost de Vries, Alex J. Poulton, Jeremy R. Young, Fanny M. Monteiro, Rosie M. Sheward, Roberta Johnson, Kyoko Hagino, Patrizia Ziveri, Levi J. Wolf

**Affiliations:** 1 BRIDGE, School of Geographical Sciences, University of Bristol, Bristol, BS8 1HB UK; 2 The Lyell Centre for Earth and Marine Science, Heriot-Watt University, Edinburgh, EH14 4BA UK; 3 Department of Earth Sciences, University College London, London, WC1E 6BS UK; 4 Institute for Geosciences, Goethe-University Frankfurt, Frankfurt am Main, 60438 Germany; 5 ICTA-UAB, Universitat Autònoma de Barcelona, Barcelona, 08193 Spain; 6 Marine Core Research Institute, Kochi University, Nankoku, 783-8502 Japan; 7 Catalan Institution for Research and Advanced Studies (ICREA), Barcelona, 08010 Spain

**Keywords:** Biogeography, Biodiversity, Microbial ecology

## Abstract

Coccolithophores are marine calcifying phytoplankton important to the carbon cycle and a model organism for studying diversity. Here, we present CASCADE (**C**occolithophore **A**bundance, **S**ize, **C**arbon **A**nd **D**istribution **E**stimates), a new global dataset for 139 extant coccolithophore taxonomic units. CASCADE includes a trait database (size and cellular organic and inorganic carbon contents) and taxonomic-unit-specific global spatiotemporal distributions (Latitude/Longitude/Depth/Month/Year) of coccolithophore abundance and organic and inorganic carbon stocks. CASCADE covers all ocean basins over the upper 275 meters, spans the years 1964-2019 and includes 33,119 gridded taxonomic-unit-specific abundance observations. Within CASCADE, we characterise the underlying uncertainties due to measurement errors by propagating error estimates between the different studies. This error propagation pipeline is statistically robust and could be applied to other plankton groups. CASCADE can contribute to (observational or modelling) studies that focus on coccolithophore distribution and diversity and the impacts of anthropogenic pressures on historical populations. Additionally, our new taxonomic-unit-specific cellular carbon content estimates provide essential conversions to quantify the role of coccolithophores on ecosystem functioning and global biogeochemistry.

## Background & Summary

Coccolithophores are marine phytoplankton and important calcifiers, impacting the ocean carbon cycle through the production of calcite (inorganic carbon)^1–3^ and primary production (organic carbon, ca. 2 to 10% of global PP^4^; and up to 40% regionally^5^). However, it is still unclear how climate change will impact coccolithophores due to many uncertainties regarding their ecology and global carbon stocks. Several issues predicate this problem: (1) *in situ* observations of coccolithophores are in cellular concentrations and need to be converted into carbon content to infer their climatic impacts; (2) conversion of abundance into carbon content generally relies on laboratory measurements, which are sparse for coccolithophores because most species are hard to culture; (3) there are uncertainties associated with measurement error and natural variability in cell size and cellular organic and inorganic contents; and (4) *in situ* observations consist of different studies which have to be extracted from multiple sources, and might use different synonyms to refer to the same taxonomic unit.

Here, we provide new and improved estimates of cell size and cellular Particulate Organic Carbon (POC) and Particulate Inorganic Carbon (PIC) contents for 139 coccolithophore taxonomic units. For this, we collated direct measurements from *in situ* water samples from the literature and developed novel allometric functions of cellular POC and PIC based on cell size. We also compiled a extensive global abundance dataset, which includes 33,119 gridded observations and covers all ocean regions and seasons over the 1965-2019 period for the same 139 coccolithophore taxonomic units. Combining cell size and carbon content estimates with the abundance dataset, we calculated the global spatial-temporal distribution (Latitude, Longitude, Depth, Month and Year) of water-column taxonomic-unit-specific PIC and POC stocks for the 139 coccolithophore taxonomic units. To ensure data quality, we thoroughly checked for errors associated with taxonomic unit synonyms and misspellings for all datasets. We only included methods that resolve individual coccolithophore taxonomic units for the abundance dataset and excluded methods that only measured total coccolithophore abundances. Besides, the dataset includes water-column abundance data previously not readily available because they were only included in hard-to-access formats such as supplemental PDF tables, notebooks and floppy disks. Finally, we carefully quantified uncertainties in the cellular POC and PIC content estimates through sample reconstruction and allometric regression error propagation.

This approach significantly improves on previous carbon content and stock estimates by (1) expanding and improving coccolithophore cell size estimates provided in MAREDAT^6^, (2) re-evaluating the allometric scaling of coccolithophore POC content with cell size using a much larger dataset (42 vs 9 observations included in a previous coccolithophore-specific allometric scaling estimate^7^), (3) estimating a novel allometric scaling function for PIC based on 961 measurements from 56 taxonomic units, and (4) providing the first global spatial-temporal distribution estimate of taxonomic-unit-specific coccolithophore PIC distributions.

The overall dataset is valuable for training species distribution models and validating mechanistic ecosystem models, which are needed to estimate coccolithophore contributions to the global carbon cycle and the impact of climate change. The dataset can also help study coccolithophore diversity and drivers of their distributions. The long period (1965-2019) covered by our dataset also makes it an excellent tool for investigating historical anthropogenic impacts on coccolithophore ecology. Finally, our dataset can help identify gaps in the spatial and temporal coverage of coccolithophore abundance, size, and carbon content, which can guide future sampling efforts.

## Methods

**Fig. 1:**
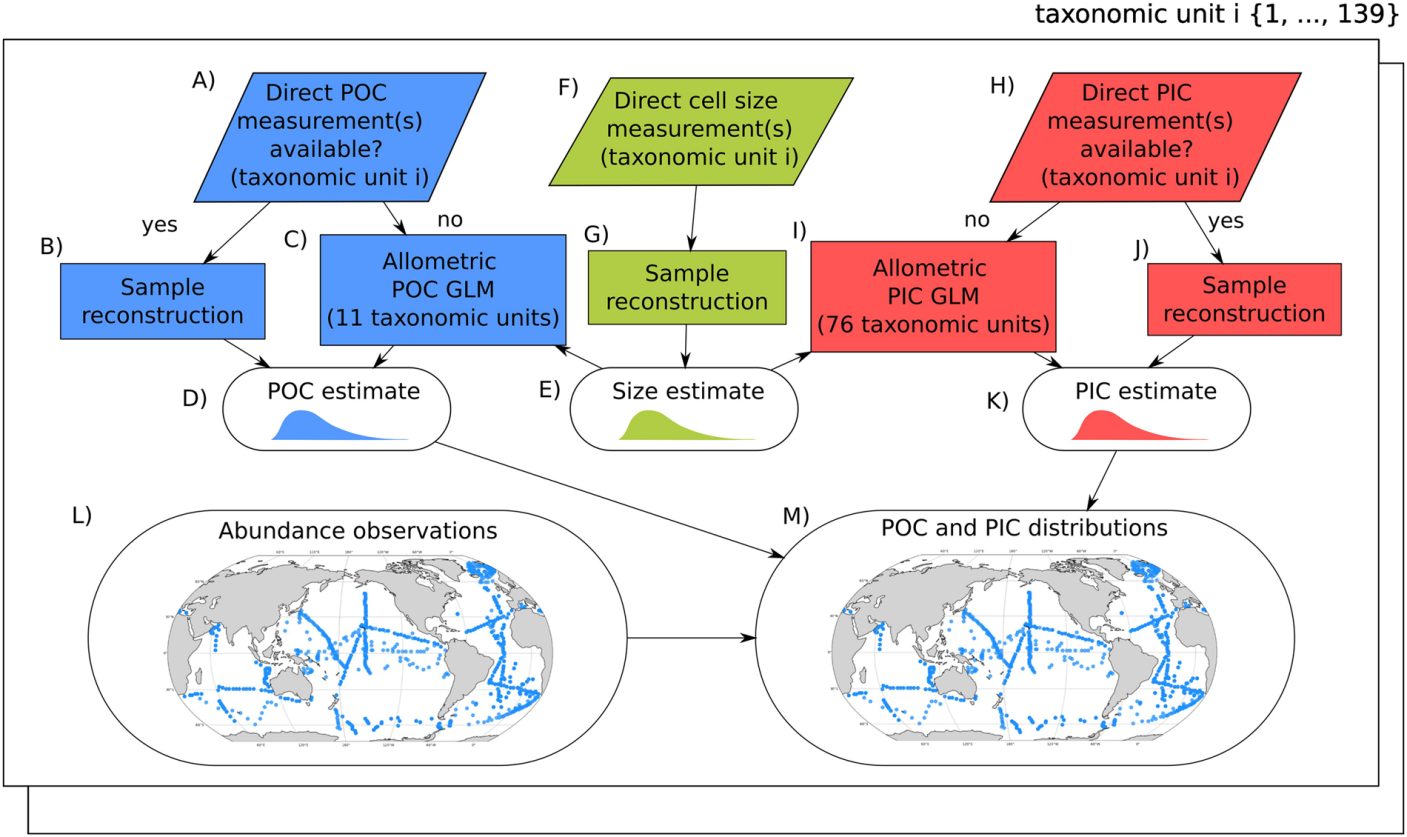
Graphical Abstract: An overview of CASCADE dataset’s pipeline. We compiled taxonomic-unit-specific cell size and cellular organic carbon (POC) and inorganic carbon (PIC) contents for 139 coccolithophore taxonomic units. We then combined them with a global abundance observational dataset to create a gridded data product (Latitude/Longitude/Depth/Month/Year) of the global distributions of taxonomic-unit-specific coccolithophore carbon stocks. The pipeline consists of the following steps for a given taxonomic unit (*i*. (**A**) Direct POC laboratory measurements of taxonomic unit *i* are compiled from the literature. (**B**) If POC measurements are available for taxonomic unit *i*, measurements are statistically reconstructed to create a merged estimate of POC content of taxonomic unit *i*. (**C**) If direct measurements are unavailable for taxonomic unit *i*, an allometric Generalised Linear Model (GLM) based on cell size and POC contents of all taxonomic units with observations is used to estimate taxonomic unit *i*’s cellular POC content from its merged cell-size distribution (**E**). The merged cell-size estimate of taxonomic unit *i* is created by compiling cell-size estimates of taxonomic unit *i* (**F**), which are then statistically reconstructed to propagate the error distributions of measurements from different studies (**G**). For cellular PIC content estimates, a similar process is applied. (**H**) PIC measurements are conducted and compiled from the literature. (**J**) If direct measurements are available for taxonomic unit *i*, its PIC measurements are statistically reconstructed and used to create a merged estimate of its PIC content (**K**). If no direct estimates are available for taxonomic unit *i*, an allometric Generalised Linear Model (GLM) based on cell size and PIC contents of all taxonomic units with observations is used to estimate taxonomic unit *i*’s cellular PIC content from its merged cell-size estimate (**E**). Finally, global spatial-temporal abundances of taxonomic unit *i* are compiled (**L**) and then converted into organic carbon (**D**) and inorganic (**K**) stocks, providing a global distribution of cellular carbon stocks for taxonomic unit *i* (**M**). This pipeline is then applied for each taxonomic unit (139 in total).

The dataset of global coccolithophore abundance and associated carbon stocks was created by combining (1) new estimates of taxonomic-unit-specific cell size and cellular POC and PIC contents for different coccolithophore taxonomic units from field samples and (2) a new compilation of global coccolithophore abundance observations from water-column samples. The sources of these data and data processing used to create the final dataset are summarised in Fig. 1 and detailed below.

We compiled global coccolithophore abundance observations from water-column samples and estimated taxonomic-unit-specific cell size, POC and PIC contents for taxonomic units with at least 20 abundance observations (139 out of 226 taxonomic units). We did not compile abundances for rarer taxonomic units (<20 observations) because their impact on the carbon cycle is likely small, and there is high uncertainty in estimating their cell sizes with so few measurements.

**Fig. 2:**
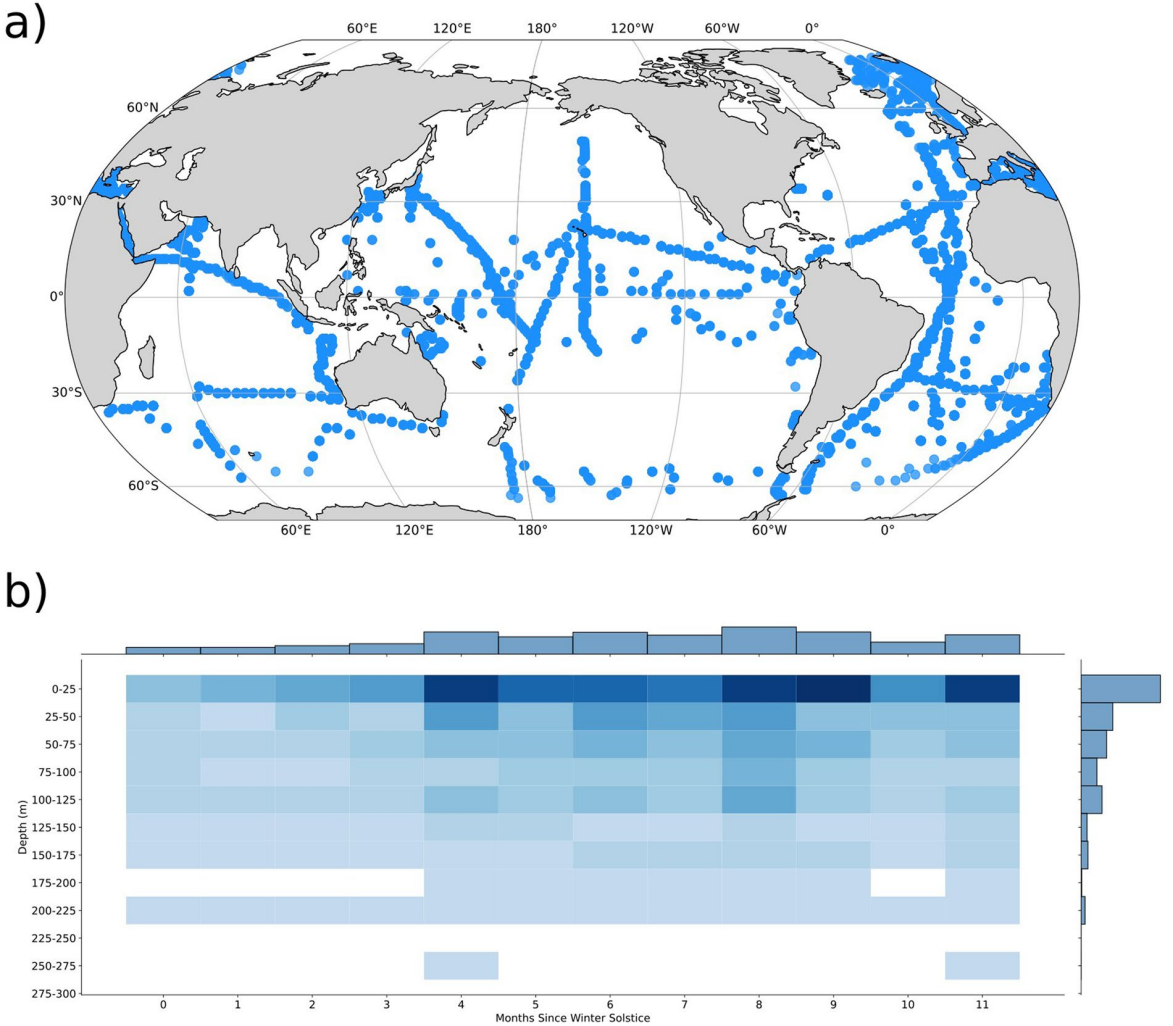
Abundance sample distribution. (**b**) Latitude and Longitude; (**b**) Depth and time. Note that most samples are in summer months and in the top 25m of the water column.

**Fig. 3:**
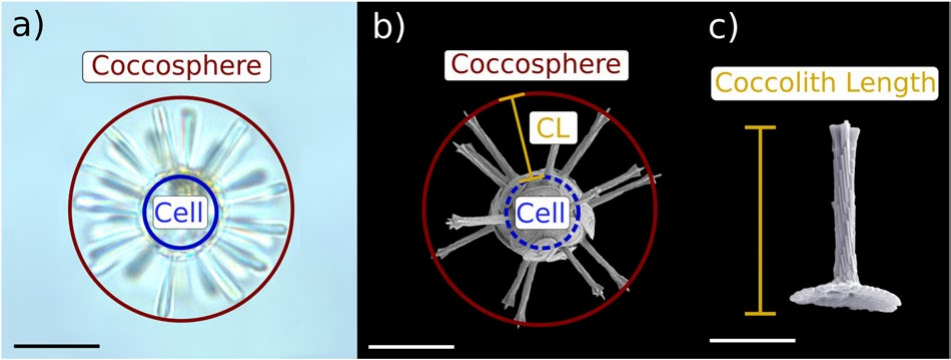
Comparison of cell size and coccosphere size for the taxonomic unit *Rhabdosphaera clavigera* HET. Coccolithophore cells (blue circles) are covered in coccoliths, forming a larger coccosphere (red circles). (**a**) By measuring the difference between coccosphere and cell size using light microscopy, cell sizes can be estimated from SEM images by measuring coccosphere size and applying a conversion factor (**b**). (**c**) Alternatively, if LM images are unavailable, SEM measurements of coccolith length (CL, orange line) can be used to estimate cell size by subtracting twice the coccolith length from the coccosphere diameter. SEM and LM images of *R. clavigera* HET coccospheres and coccolith by Jeremy Young.

**Fig. 4:**
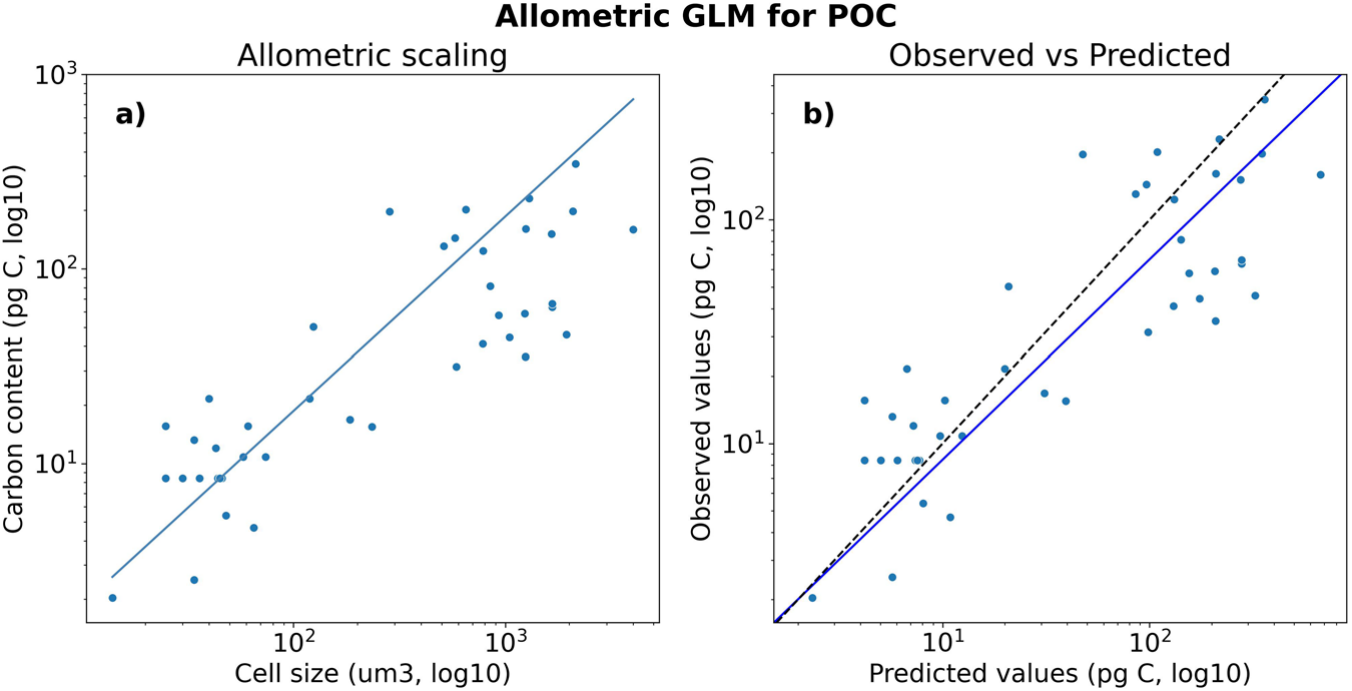
GLM allometric scaling of coccolithophore cellular POC content (**a**) Fitted GLM allometric scaling (line) compared to observations (points). (**b**) Comparison between the GLM predicted and observed cellular POC content. The black line represents a 1:1 prediction between observed and fitted values. Note that x- and y-axis are on a log10 scale.

**Fig. 5:**
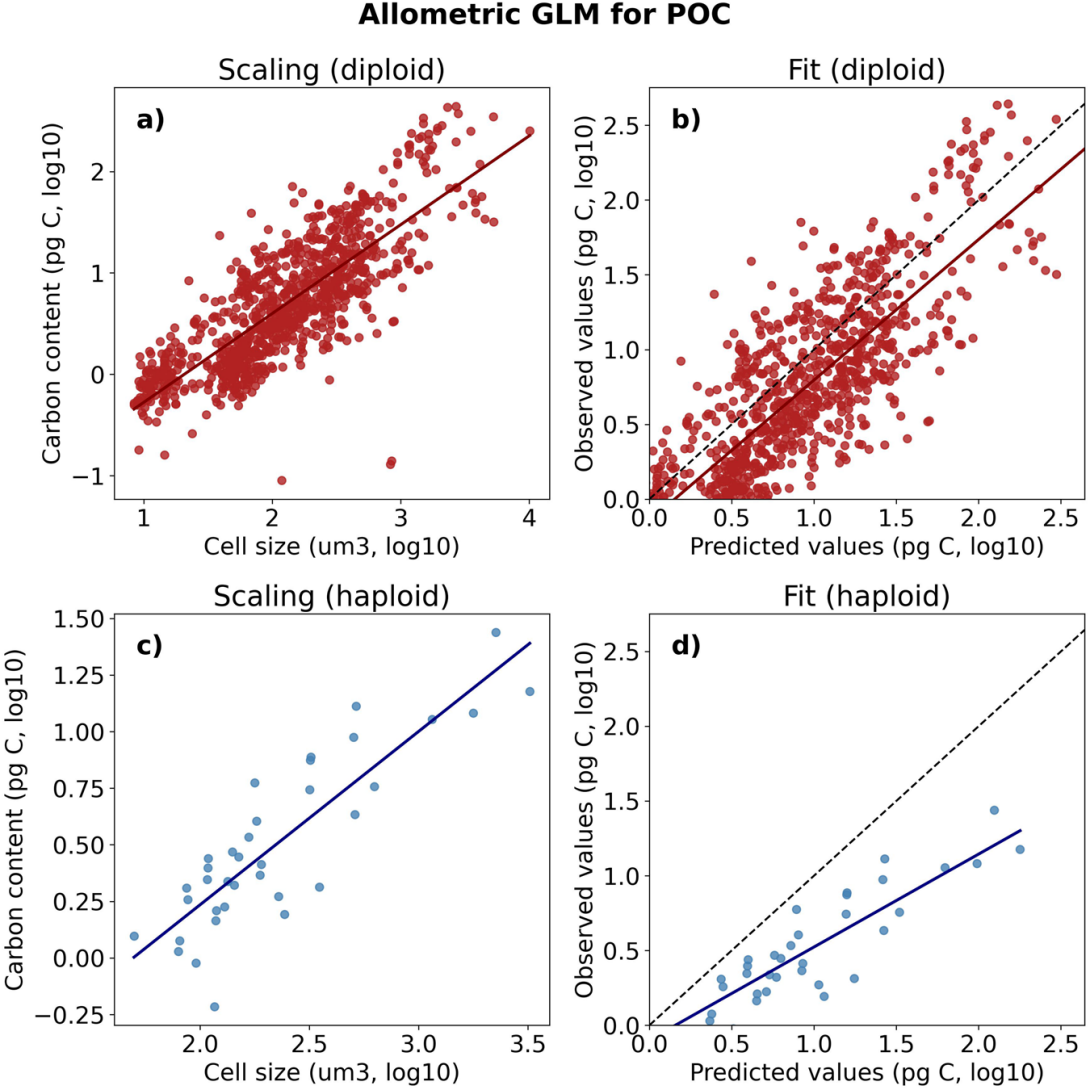
GLM allometric scaling of coccolithophore cellular PIC content per life stages. (**a**) Fitted GLM allometric scaling (red line) over trained data (red points) for the coccolithophore diploid morphotypes. (**b**) Comparison between the GLM fitted and observed cellular PIC content for diploid morphotypes (**c**) Fitted GLM allometric scaling (blue line) over trained data (blue points) for the coccolithophore haploid morphotypes. (**d**) Comparison between the GLM fitted and observed cellular PIC content for haploid morphotypes. Note that x and y-axis are on a log10 scale.

**Fig. 6:**
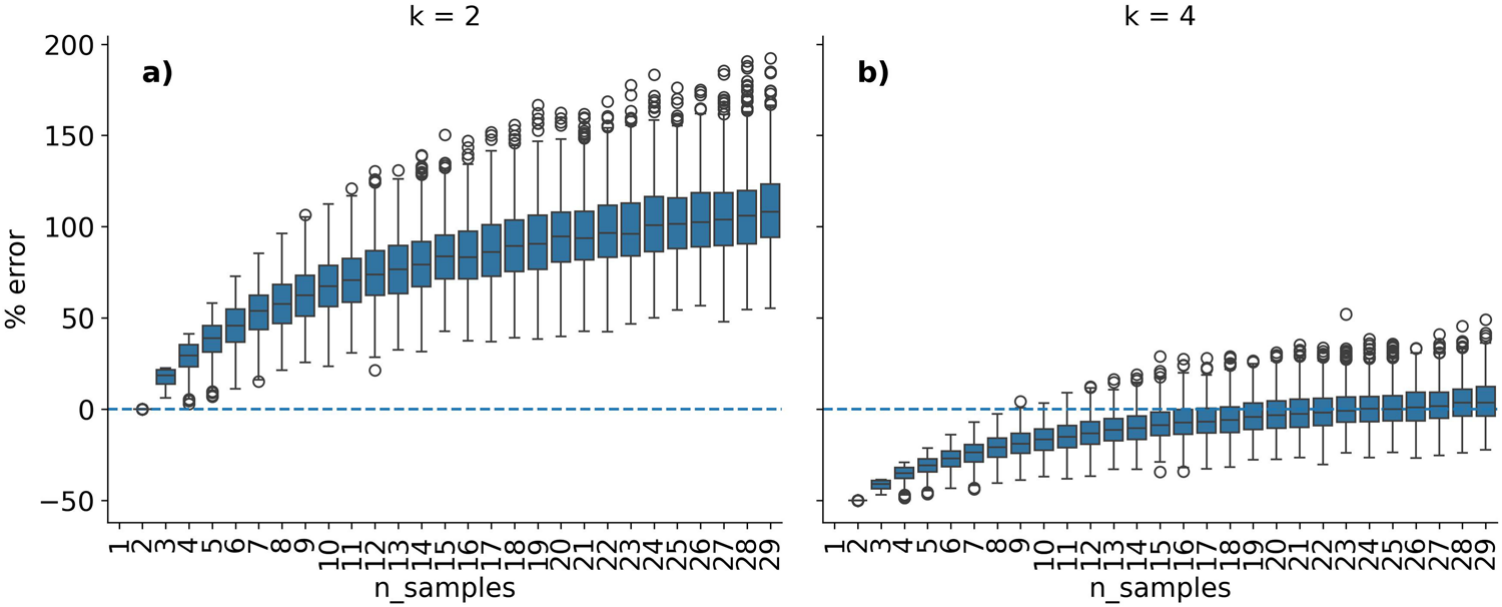
The percentage error of SD when estimated based on min-max values. (**a**) Percentage error of estimated SD based on min-max when values are divided by two. (**b**) Percentage error of estimated SD based on min-max when values are divided by four. The horizontal line is 0 percentage error; values above this line represent over-estimates of SD (conservative estimates), while values below this line represent under-estimates of SD. Note that when min-max values are divided by two (**a**), SD is never underestimated, even when the sample size is 1.

**Fig. 7:**
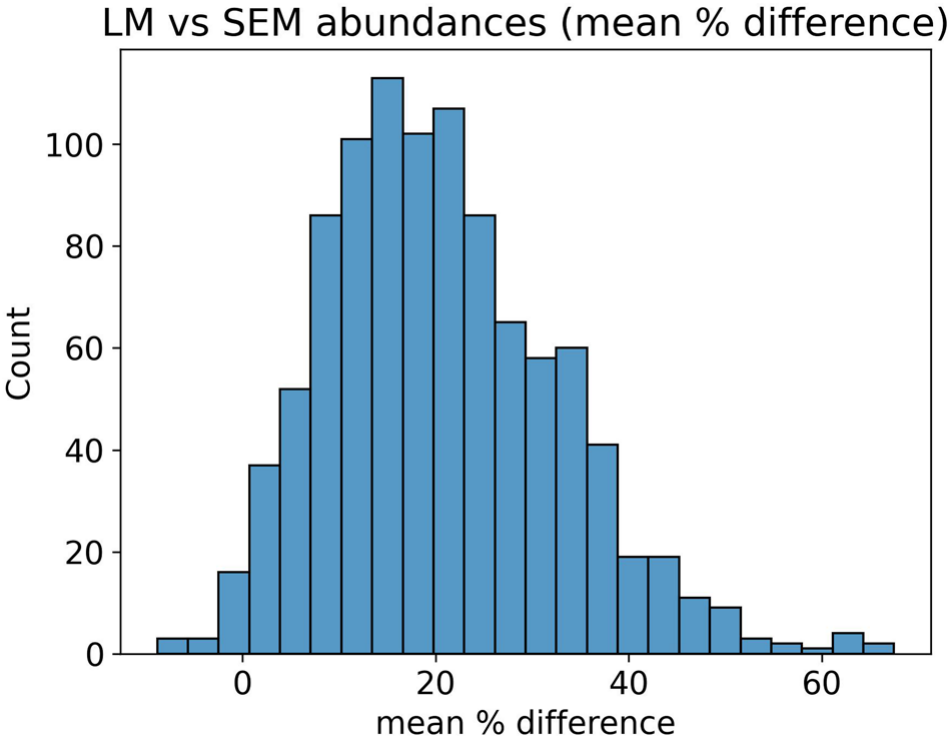
Comparison of abundance estimates from *in situ* observations counts of taxonomic units collected from the same water sample using SEM or LM. Data was acquired from Bollman *et al*.^70^ and re-analysed using Bayesian bootstrapping of relative % difference values for each sample and taxonomic unit. Note that we merged sub-species and varieties and excluded non-taxonomic-unit-specific counts (e.g. undefined *Syracosphaera* sp.).

**Fig. 8:**
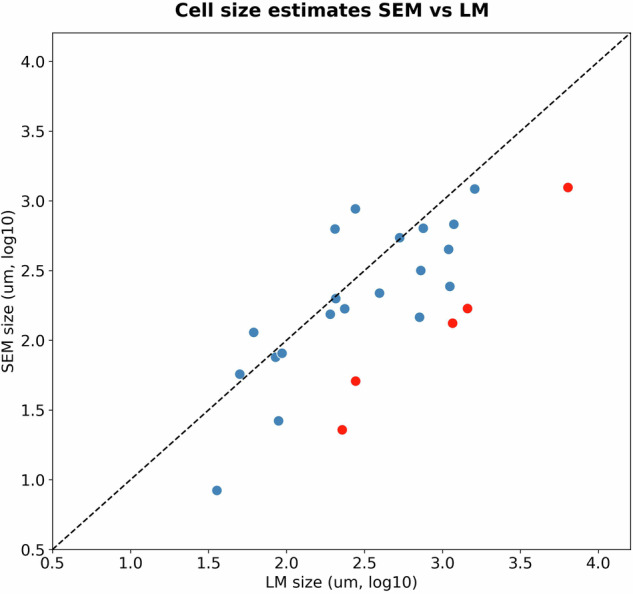
Agreement between light microscopy (LM) and scanning electron microscopy (SEM) cell size estimates. For LM cell size was measured directly, while for SEM cell size was estimated based on coccosphere size and coccolith thickness. For taxonomic units where the disagreement was greater than 5-fold (red points), only LM estimates were used. Such taxonomic units included: *Algirosphaera robusta* HET, *Helicosphaera pavimentum* HET, *Rhabdosphaera clavigera* HET, *Syracosphaera mediterranea* HOL *wettsteinii* type, and *Umbilicosphaera sibogae* HET.

Cell size estimates were compiled from the literature^6–16^ and measured using Light Microscopy (LM)^17^ and Scanning Electron Microscopy (SEM)^18^ for all 139 taxonomic units. Cellular POC estimates were compiled from laboratory observations of POC content for 11 taxonomic units from 7 studies^7,8,10,12,15,19,20^. Finally, we compiled cellular PIC content estimates for 3 taxonomic units from 2 studies^21,22^ and provide morphometric *in situ* measurements for 56 taxonomic units from the 14th cruise of the Atlantic Meridional Transect (AMT) programme (April-June 2004^18,23^).

We used a model of allometric scaling for coccolithophore taxonomic units without direct measurements of cellular POC and PIC contents, converting their cell size estimates into cellular carbon content. Specifically, we modelled allometric scaling using Generalized Linear Models (GLMs), a class of linear regression models that can explicitly characterise the distribution of the measurement data even when the outcome is not normally distributed.

We also accounted for any underlying uncertainties at each step in our analysis by propagating errors. We achieved this for measurements such as cell size and cellular POC and PIC contents by sampling the data based on the standard error of the mean and bootstrapping the resulting distributions. We propagated the error using simulations obtained from the GLMs for allometric estimates. More details of these error propagation methods are provided below.

### Taxonomy, misspellings, and synonyms

We followed the latest coccolithophore taxonomic definitions provided by NannoTax3 (www.mikrotax.org/- last accessed January 2024)^24^. We considered life cycle phases (e.g. HET and HOL) but did not consider finer taxonomic resolution than species level (i.e., no classification of sub-species, morphospecies, morphotypes, or varieties in the dataset), as they are poorly resolved in our *in situ* observation compilation and can be relatively subjective. All records in our dataset were thoroughly checked for taxonomic unit misspellings and synonyms to ensure consistent, accurate and up-to-date taxonomy. These synonyms and misspellings are provided as a YAML file in the data archive^25^.

We excluded *Reticulofenestra sessilis* HET from our dataset, as this species occurs exclusively in association with a centric diatom of the genus Thalassiosira^26^, which makes estimating POC and PIC stock for this organism challenging.

Additionally, we excluded rare taxonomic units (88 taxonomic units^25^, <20 global occurrences) because they are highly unlikely to form a significant contribution to the carbon cycle or diversity calculations (even regionally). Furthermore, we lack cellular PIC and POC estimates for rare taxonomic units due to their infrequent occurrence.

#### Life cycle associations and coccolith morphology

Coccolithophores have a distinct ‘haplo-diplontic’ life cycle, which allows them to grow and divide in two different life cycle phases (‘haploid’ and ‘diploid’)^27–29^. These two life cycle phases are morphologically distinct, with more heavily calcified diploid life cycle phases generally utilizing a heterococcolith (‘HET’) morphologies and more lightly calcified haploid cells utilizing naked, holococcolith (‘HOL’), ceratolith (‘CER’), or polycrater (‘POL’) morphologies^8,30–32^. Furthermore, haploid and diploid life cycle phases for a given species tend to have similar cell sizes^8,31,33^.

While there are some notable exceptions to these trends, as in some cases naked morphologies have been observed to be diploid^34^, and some diploid morphotypes can be lightly calcified^9^, we can nonetheless use this information in our analysis to make better estimates of cell sizes and PIC contents for taxonomic units for which we do not have direct measurements. We thus compiled coccolith type and known life cycle associations from NannoTax3^24^ for each taxonomic unit in our dataset (Supplementary Table 1).

### Propagating uncertainties

All data in our compilation have underlying uncertainties due to instrument measurement error and natural variations of cell size and cellular carbon content, which can vary between studies. Accurately capturing these uncertainties and sources of variability is important, as these are needed to determine confidence in standing stock estimates or when making inferences about ecological processes such as drivers of diversity or impacts of anthropogenic climate change. However, propagating and integrating sources of uncertainty across studies is particularly challenging when combining multiple studies with studies using different measuring conditions, populations, or instruments. As a result, each study generates different estimates with unique error distributions. Another challenge is when we rely on multiple variables to estimate the cellular carbon contents (e.g., estimating POC content based on size), we also need to propagate the uncertainties across all variable estimates.

Here, we implemented various forms of sample reconstruction to propagate uncertainties across the different methods and estimates from our multiple studies. Conceptually, the idea is to generate large sets of random values of the estimated variables (e.g., cell size). These replicates represent the potential outcomes of each study given their error distribution and allow us to model the uncertainty distribution of each study. We then combined each study’s replicated distribution set to produce merged distribution of possible measurements, representing the combined uncertainties of all different studies.

For example, imagine a given species i is measured in two observational studies, each with estimates of mean cell size and standard error of the mean (SE) in cell sizes. Study A’s mean size is 10 μm with a SE of 2 μm, while study B’s mean size is 12 μm with a SE of 3 μm. Assuming the distribution of cell sizes is normally distributed, we generate a set of cell size values for each study based on its mean and standard error of the mean. Study A’s set might be 11.6, 6.0, 9.7, while study B’s set might be 7.6, 18.0, 16.9. We can then append these two sets of numbers to create a new set 11.6, 6.0, 9.7, 7.6, 18.0, 16.9. Finally, this merged distribution can be smoothed using Bayesian bootstrapping and then used to calculate a newly merged mean and confidence interval based on percentile estimates.

In our study, we used different methods to simulate these sets of values depending on the context and type of data available. The method above (assuming a zero-truncated normal distribution) was used for direct measurements with only a mean and SE. For direct measurements where the original data was available, we used Bayesian bootstrapping for sample reconstruction instead. Bootstrapping is a type of resampling that uses random sampling by replacement to create new distributions while maintaining the original uncertainty distribution^35^. Bayesian bootstrapping is a generalised version of bootstrapping, which further allows weighting of the sub-samples to create smoother distributions^36^. We used Bayesian bootstrapping if possible because the method preserves the original data’s underlying distribution without making any normality assumptions while creating smoother reconstructed sample distributions. In some cases, studies only reported maximum and minimum values instead of SE; for such studies, we estimated SD using a modified version of the ‘range rule’.

1 $${\rm{SD}}=({\rm{Maximum}}-{\rm{Minimum}})/2$$

This first-order approximation assumes a normal distribution and that the reported maximum and minimum values represent one standard deviation from the mean. Based on simulation (Fig. 6a), this tends to overestimate the standard deviation even when the original sample size is small and thus is a conservative estimate. This is critical since all studies reporting only min/max values measured only a few cells^37–44^. We thus use division by two instead of the standard range rule, which assumes min and max represent two deviations from the mean (i.e. division by four)^45^, and underestimates the standard deviation if the sample size is small (Fig. 6b). SE was then estimated from the SD by assuming a sample size of three.

For direct measurements with no SE, original data or min and max values, SE was simulated by bootstrapping from normalized SEs from other measurements of the same taxonomic unit. If no other SEs were available for the same taxonomic unit, cross-study-and-cross-taxonomic-unit normalized SEs were used.

#### Uncertainty in Generalised Linear Modelling estimates

When direct measurements were unavailable, carbon content was estimated based on cell size (‘allometric estimates’). For these estimates, errors were propagated by accounting for model uncertainty of the slope used to convert cell size into cellular carbon content. We achieved this by bootstrapping of training data before fitting multiple GLMs (one for each bootstrapped sample).

#### Implementation in Python

All error propagation was conducted in Python using either Numpy^46^ (np) or Statsmodels^47^ (sm). If only mean and SD were known, np.random.normal was used, which was zero-truncated by removing values below zero using np.clip. If the original data was available, np.random.choice.dirichlet was used for Bayesian bootstrapping. To simulate GLM distributions, sm.GLM.get_distribution was used.

### Coccolithophore cell size

We compiled coccolithophore cell size estimates from the literature. The cell size component of our dataset is sourced from a range of observation and measurement types. It includes new scanning electron microscopy measurements^18^ and light microscopy measurements^17^ from plankton samples, size measurements from laboratory cultures^6–8,10–13,15,16,48,49^, and literature morphometric estimates of cell size^7^. Each measurement type has its advantages and disadvantages, which are discussed below.

Regarding observation types, laboratory cell size measurements generally benefit from more samples but are usually limited to single genetic strains of a taxonomic unit grown under a controlled range of environmental conditions. Therefore, cell size measurements from laboratory cultures do not capture the full natural variation of cell sizes that occur *in situ*, where the size distribution of taxonomic unit populations will be influenced by genetic and phenotypic diversity and a wider range of environmental conditions^50^. However, a smaller quantity of cell size measurements from plankton samples are available, and the observations are often spatially biased (similarly to the availability of abundance data). Here, we overcome some of these limitations by combining *in situ* (plankton) and laboratory measurements of cell size to estimate the overall expected distribution of cell size of individual coccolithophore taxonomic units.

Regarding measurement types, LM provides more accurate cell size measurements than SEM measurements. This is because only the coccosphere surface (i.e. including the inorganic carbon exoskeleton, the coccosphere) is observable through SEM, whereas, under LM, fine adjustments to the focal depth of the microscope allow the observer to image a cross-section of the cell that reveals the internal size of the coccosphere (approximately the diameter of the cell cytoplasm)^48^. Therefore, cell size measurements from SEM must be derived from coccosphere size measurements. In addition, both LM and SEM can be spatially and temporally biased and might thus fail to capture the full range of natural intra-specific variations. Therefore, to maximise the use of available data, we combined both SEM and LM measurements for each cell size estimate, except if there was a disagreement in median cell size 5-fold times greater between LM and SEM measurements for a given taxonomic unit, in which case only LM (as the more accurate cell size measurement) was used. Generally, there was a good agreement between LM and SEM (Fig. 8).

We also used resampling to propagate any uncertainties in the observed measurements to maintain the uncertainty distributions resulting from natural intraspecific variations in cell size and measurement errors of each input size dataset. For a detailed description, please refer to the section Uncertainty Propagation.

#### *In situ* scanning electron estimates of cell size

Extant coccolithophore cell size estimates from plankton samples were derived from a new dataset of taxonomic unit-level morphometric traits measured on legacy SEM images from 16 samples (10,665 images) from AMT-14 (zenodo.11483788)^18^. From the SEM images (zenodo.10571820)^51^, the coccosphere diameter (long axis and short axis measurement) of each intact coccosphere encountered was measured using the freeware Fuji (v1.53a)^52^. The SEM morphometric trait database includes 961 coccosphere size measurements (as equivalent spherical diameter) for 56 taxonomic units. For each coccosphere, cell size was estimated from measured coccosphere size using a taxonomic-unit-specific conversion factor that defines the percentage of coccosphere volume that is represented by cell size (Fig. 3). The conversion factor was derived from LM measurements of cell size and coccosphere size on the same coccospheres^17^. The methodology for estimated cell size from coccosphere size measurements on SEM images and related uncertainties is described in detail in the associated publication^23^.

#### *In situ* light microscopy estimates of cell size

With light microscopy, we can directly observe the internal space defined by the coccosphere, which, for most taxonomic units, can be assumed to correspond closely to the cell volume. For this study, slides from previous work that had common, well-preserved coccospheres were selected. The slides had been prepared during various research cruises, but in all cases, by filtration of seawater onto cellulosic filters of 0.2 to 0.4 μm pore size. After filtration, the filters were oven-dried, and then a portion of them was immediately mounted on a glass slide using low-viscosity optical adhesive (Norland Optical Adhesive NOA 74). Coccospheres were identified using polarising light microscopy for cell volume measurement and then imaged in cross-polars and bright fields. The saved images were measured using the image analysis program Fuji^52^. This work focused on the most common/numerically important taxonomic units but excluded Emiliania, Gephyrocapsa and Coccolithus, for which extensive data is available from the literature. This dataset can be accessed from Zenodo (zenodo.10572754)^17^.

#### Laboratory light microscopy measurements of cell size

Our compilation of published laboratory cell size estimates consists of 11 different studies^6,7,9–13,15,16,49,53^. We checked the method used for each study and converted diameter values to volume where appropriate. We did not include studies that only measured coccosphere size because converting such estimates to cell size adds significant uncertainty due to variations in coccosphere thickness. A concatenated dataset of the 11 studies can be found on Zenodo (zenodo.12794780)^25^.

#### Literature morphometric estimates of cell size

For taxonomic units with no direct cell size measurements but where coccosphere size measurements were available in the literature, we similarly converted coccosphere size to an estimate of cell size by subtracting a taxonomic-unit-specific estimate of coccolith thickness or coccosphere thickness from coccosphere diameter (Fig. 3). These size conversion factors were taken from previous literature^7^ or newly estimated for this study for taxonomic units not included in other datasets^24^.

We have not used the literature morphometric cell size estimates from MAREDAT^6^, which estimated cell dimensions for all extant coccolithophore taxonomic units by assuming a fixed ratio of 0.6 between coccosphere and cell volume (i.e. assuming that cell volume is consistently 60% of coccosphere volume). This assumption introduces significant uncertainties, as the ratio between coccosphere and cell size can vary from 0.3 to 0.9 between taxonomic units^6^. In extreme cases, this leads to 3 to 8-fold differences between estimates of cell volume and, subsequently, POC cellular content.

Therefore, we followed the approach described in Villiot *et al*.^7^ for our cell size estimates. Briefly, we compiled coccosphere diameter (or long axis and short axis diameter measurements for non-spherical taxonomic units) and coccolith thickness from the literature. Cell dimensions were then estimated by subtracting twice the coccolith thickness from the coccosphere diameter (Fig. 3b-c). Finally, cell volume was calculated by assuming a spherical or prolate spheroid coccosphere shape, depending on the taxonomic unit (Table 1). All taxonomic units were assumed to have a spherical coccosphere except for *Syracosphaera aurisinae* HET and *Calciopappus caudatus* HET, which were assumed to have a prolate spheroid coccosphere.

#### HOL life cycle cell size

Since haploid and diploid life cycle phases for a given taxonomic unit tend to have similar cell sizes^8,31,33^, if available, the associated HET life cycle phase(s) values were used to estimate the cell size of the HOL phase lacking size measurements. A mean of associated HET life cycle phases was used for HOL morphotypes with multiple HET life cycle phases. For example, for the HOL life cycle phase *Helladosphaera cornifera* HOL estimates from *Syracosphaera nodosa* HET^30^ and *Syracosphaera noroitica* HET^54^ were used, while for the HOL life cycle phase *Sphaerocalyptra quadridentata* HOL estimates from *Algirosphaera robusta* HET^55–57^ and *Rhabdosphaera clavigera* HET^37^ were used.

The full list of life cycle associations utilized in this study can be found in Supplementary Table 1 and is provided as a YAML file in the Zenodo data archive (zenodo.12794780)^25^.

### Cellular POC content

#### Laboratory combustion estimates of POC

Direct measurements of cellular POC were compiled from previously published laboratory data. We compiled all studies with direct measurements of coccolithophore cellular POC content (11 studies)^6–8,10–13,15,16,48,49^. This included combustion estimates, which were treated with an acid solution to remove PIC. We excluded studies that utilised combustion but did not specify an acid treatment, as it was unclear if such studies measured POC or a net cellular carbon content (PIC and POC) instead^16^. We also excluded studies that utilised wet oxidation treatments^13^.

Overall, our cellular POC data compilation included 11 taxonomic units and 42 observations. If available, for each study, we also compiled cell size estimates which were used for our allometric GLM scaling (7 studies, 11 taxonomic units, and 42 observations). The POC dataset is included as part of the Zenodo data archive (zenodo.12794780)^25^.

Where available, direct measurements of cellular POC were resampled to create a merged estimate of cellular POC content. For a detailed description, please refer to the section Uncertainty Propagation.

#### Allometric scaling and cellular POC content estimation

For coccolithophore taxonomic units which did not have direct cellular POC content measurements, we applied an allometric scaling function to estimate the cellular POC content from cell size. For this, we created a new allometric scaling function by fitting a Generalised Linear Model (GLM) to a significantly larger number of coccolithophore observations from previous studies (42 vs 4 included in Menden-Deuer and Lessard^58^ and 9 in Villiot *et al*.^7^). GLMs improve data fit compared to traditional linear regressions because GLMs can 1) constrain the predicted POC to be strictly positive by defining a Gamma distribution and 2) retain the training data’s error distributions^59^.

We used all available data points from our compilation (42 data points) representing 11 taxonomic units to fit a GLM allometric scaling for cellular POC content. We applied this GLM scaling to estimate the cellular POC content of taxonomic units with no direct estimates. The model has a mean prediction error of 77 pg C per cell and a root mean squared prediction error of 137 pg C per cell. Relative to the observed mean value of 67 pg C per cell, this constitutes relative MAE values of 113% and relative RMSE values of 200% (Fig. 4).

We compared the performance of the Gamma distributed GLM model with the more commonly used Gaussian distributed linear regression^7,58^. The Gaussian distributed linear regression models did not perform as well with lower Cox and Snell pseudo-R-squared values (0.52 vs 0.69) and higher Akaike Information Criterion (AIC) information loss (539 vs 399) and was thus not used.

The allometric scaling slope of our GLM compares well with previous literature, with a slope of 0.72 when cell size and POC are plotted on a log10 scale (Fig. 4). This estimated slope falls between the previously reported scaling slope of 0.7 reported for coccolithophores^7^ and 0.95 previously reported for protists^58^.

### Cellular PIC content

We compiled estimates of cellular PIC from the literature. For taxonomic units with direct measurements, we resampled these observations to determine the mean and standard deviation in cellular PIC content for each measured taxonomic unit. To estimate PIC of taxonomic units with no direct measurements, we used an GLM allometric scaling of cellular PIC content, which we fitted based on the observed values.

#### *In situ* scanning electron microscope estimates of PIC

56 of our taxonomic units have direct cellular PIC estimates based on Sheward *et al*.^23^. Underlying assumptions and methodological descriptions of these estimates are provided in full detail in Sheward *et al*.^23^. In brief, Sheward *et al*.^23^ estimated the cellular PIC content of 56 taxonomic units using morphometrics. This method combines estimates of coccolith PIC content based on coccolith morphology and size^60^ and then multiplies this estimate by the number of coccoliths observed per coccosphere to estimate the cellular PIC content. Coccolith size measurements and cellular counts were made using SEM images collected during the Atlantic Meridional Transect 14 cruise (28 April to 1 June 2004), which covered 47.03° S to 49.25° N^61^. The images (zenodo.10571820)^51^ and resulting dataset (zenodo.11483788)^18^ can be found on Zenodo.

#### Laboratory morphometric estimates of PIC

We also compiled morphometric estimates of cellular PIC content from the literature, which were available for three different taxonomic units: *Coccolithus pelagicus* HET, *Calcidiscus leptoporus* HET and *Helicosphaera carteri* HET^21,22^. For *Calcidiscus leptoporus* HET, two sub-species of *Calcidiscus leptoporus* HET were measured: *Calcidiscus leptoporus* subsp. *quadriperforatus* HET and *Calcidiscus leptoporus* subsp. *leptoporus* HET. While some authors consider these subspecies to be a separate species^62–64^, *C. leptoporus* HET morphotypes are hard to distinguish morphologically and are not distinguished in our *in situ* abundance compilation. We thus considered both *Calcidiscus leptoporus* subsp. *quadriperforatus* HET and *Calcidiscus leptoporus* subsp. *leptoporus* HET as a single taxonomic unit here. The original datasets (PANGAEA.836841)^21^ and (PANGAEA.865403)^22^ can be found on PANGAEA.

#### Laboratory combustion estimates of PIC

We did not include laboratory combustion estimates of PIC. Such measurements include discarded coccoliths and thus overestimate cellular PIC. While the contribution of discarded coccoliths depends on the taxonomic unit, since, for example, for the taxonomic unit *Calcidiscus leptoporus* HET the number of discarded coccoliths is low^65^, the ratio between attached and discarded coccoliths can be over 6x for *Emiliania huxleyi* HET if the culture is not maintained at optimum conditions^66^.

#### PIC allometric scaling

For taxonomic units which did not have direct cellular PIC content measurements, we followed a similar approach taken for POC, where we used a GLM allometric scaling function to estimate cellular PIC content from cell size. However, we also include the life cycle phase as a variable to estimate PIC. Life cycle phases strongly influence cellular PIC contents, with haploid morphologies generally containing much lower cellular PIC contents^8,30–32^. To define the life cycle phase in the model, heterococcolith (HET), and Nannolith (NANO) life cycle phases were labelled as ‘diploid’ while ceratolith (CER), holococcolith (HOL) and polycrater (POL) were labelled as ‘haploid’. While HET cells might not be strictly diploid and HOL, CER and POL cells might not be strictly haploid^34^, for simplicity, and since it will not influence PIC estimates, we refer to them as such here. Life cycle phases were included by one-hot-encoding the variables using the Pandas^67^pd.get_dummies function.

We fitted the GLM allometric scaling for the cellular PIC content based on 961 data points (925 diploid and 36 haploid) (Fig. 5). The models were used to estimate the cellular PIC content of 76 taxonomic units. The GLM PIC model has mean prediction error of the model is 13 pg C per cell, with a root mean squared prediction error of 34 pg C per cell. Relative to the observed mean value of 16 pg C per cell, this constitutes relative MAE values of 77% and relative RMSE values of 211%.

### *In situ* abundance observations

We compiled water-column observations of coccolithophore abundance from the literature (6,166 samples from 62 studies, Table 2 and Table 3, some of which have previously been included in coccolithophore abundance compilations^6,28^, which were extracted from their respective PANGAEA data archives (PANGAEA.785092)^68^ and (PANGAEA.922933)^69^. The observations included in our coccolithophore abundance compilation are restricted to methods that can resolve taxonomic unit identity: Scanning Electron Microscopy (SEM) and light microscopy (brightfield and polarised, LM). We excluded observations from methods that cannot resolve taxonomic units, such as flow cytometric measurements and data records, where methods used for taxonomic identification were unspecified Table 1.

**Table 1:** Species for which cell size was estimated based on literature morphometric estimate of coccosphere size and coccolith thickness.

Species	Vol (mean)	Vol (SD)	Cell shape	Ref.	obs.
*Alisphaera gaudii* POL	134.2	79.7	Spherical	Cros and Fortuno^37^	26
*Calciopappus caudatus* HET	23.4	3.9	Prolate spheroid	Gaarder and Ramsfjell^38^	177
*Calicasphaera blokii* HOL	33.5	—	Spherical	Cros and Fortuno^37^	32
*Calicasphaera concava* HOL	20.6	—	Spherical	Cros and Fortuno^37^	27
*Calyptrosphaera sphaeroidea* HOL	346.4	186.2	Spherical	Cros and Fortuno^37^	81
*Helicosphaera* HOL *confusus type*	472.3	181.0	Spherical	Cros and Fortuno^37^	27
*Helladosphaera pienaarii* HOL	190.0	99.5	Spherical	Norris^39^	25
*Ophiaster formosus* HET	51.5	25.0	Spherical	Cros and Fortuno^37^	187
*Ophiaster hydroideus* HET	44.7	3.0	Spherical	Cros and Fortuno^37^	490
*Pappomonas sp. type 5* HET	144.3	16.5	Spherical	Cros and Fortuno^37^	31
*Sphaerocalyptra adenensis* HOL	70.0	31.0	Spherical	Cros and Fortuno^37^	40
*Syracosphaera aurisinae* HET	942.7	150.0	Prolate spheroid	Lecal-Schlauder^40^	91
*Syracosphaera borealis* HET	81.7	19.3	Spherical	Okada and McIntyre^41^	45
*Syracosphaera reniformis* HET	59.8	19.0	Spherical	Kleijne and Cros^42^	39
*Syracosphaera squamosa* HET	11.7	2.5	Spherical	Kleijne and Cros^42^	48
*Syracosphaera strigilis* HET	91.7	37.1	Spherical	Cros and Fortuno^37^	24
*Wigwamma antarctica* HET	65.4	—	Spherical	Thomsen *et al*.^43^	41
*Zygosphaera marsilii* HOL	174.4	37.6	Spherical	Borsetti *et al*.^44^ Cros and Fortuno^37^	45

Diameter (or length and width for prolate spheroid morphologies) was estimated by subtracting twice the coccolith thickness from coccosphere length. After this, cell volume (Vol) was estimated based on the cell shape. For these species’ estimates, SDs were unavailable and, if possible, estimated by assuming the min and max values represented the 68% quantiles: SD = (max-min)/2.

**Table 2:** Scanning Electron Microscopy (SEM) studies compiled as part of the abundance compilation.

Reference	Method	Samples	Survey Period
Andruleit *et al*.^86^	SEM	71	1993–1993
Andruleit *et al*.^87^	SEM	21	2000–2000
Andruleit *et al*.^88^	SEM	45	1999–1999
Andruleit *et al*.^6^^†^	SEM	48	2001–2002
Baumann *et al*.^78^	SEM	98	1987–1995
Baumann *et al*.^89^	SEM	34	1993–1993
Boeckel *et al*.^90^	SEM	56	1998–2000
Bonomo *et al*.^91^	SEM	92	2008–2008
Bonomo *et al*.^92^	SEM	40	2012–2012
Bonomo *et al*.^93^	SEM	120	2009–2009
Bonomo *et al*.^94^	SEM	301	2010–2014
Bonomo *et al*.^95^	SEM	133	2016–2016
Cepek *et al*.^96^	SEM	33	1993–1993
Cerino *et al*.^72^	SEM	77	2011–2013
Charalampopoulou *et al*.^97^	SEM	59	2008–2008
Charalampopoulou *et al*.^98^	SEM	89	2009–2009
Cortes *et al*.^81^	SEM	171	1994–1996
Cros *et al*.^99^	SEM	113	1996–1996
D’Amario *et al*.^100^	SEM	44	2013–2013
Daniels *et al*.^32^	SEM	19	2012–2012
Dimiza *et al*.^57^	SEM	190	2001–2004
Dimiza *et al*.^101^	SEM	99	2001–2013
Dimiza *et al*.^102^	SEM	12	2016–2016
Eynaud *et al*.^103^	SEM	44	1995–1995
Giraudeau *et al*.^104^	SEM	146	2014–2014
Godrijan *et al*.^71^	SEM	23	2008–2009
Guerreiro *et al*.^105^	SEM	108	2010–2010
Guptha *et al*.^106^	SEM	12	1992–1992
Hagino *et al*.^107^	SEM	225	1964–1992
Haidar *et al*.^80^	SEM	193	1991–1994
Karatsolis *et al*.^108^	SEM	68	2013–2014
Keuter *et al*.^109^	SEM	38	2018–2019
Keuter *et al*.^110^	SEM	196	2017–2019
Kinkel *et al*.^111^	SEM	40	1994–1996
Kleijne 1985^112–114^^‡^	SEM	100	1985–1985
Luan *et al*.^115^	SEM	52	2013–2013
Malinverno *et al*.^116^	SEM	72	1997–1997
Malinverno *et al*.^117^	SEM	13	2005–2005
Okada *et al*.^75^	SEM	226	1968–1969
Oviedo *et al*.^14^	SEM	81	2011–2012
Patil *et al*.^118^	SEM	48	2010–2010
Poulton *et al*.^61^	SEM	137	2003–2005
Saavedra-Pellitero *et al*.^119^	SEM	109	2010–2010
Schiebel *et al*.^120^	SEM	47	1995–1997
Schiebel *et al*.^121^	SEM	47	2004–2004
Silver^6^*	SEM	13	2004–2004
Smith *et al*.^122^	SEM	25	2011–2012
Supraha *et al*.^123^	SEM	63	2013–2013
Takahashi *et al*.^124^	SEM	118	1996–1996

^†^Dataset published as part of O’Brien *et al*.^6^. ^‡^Dataset resulting from Snellius-II Cruise Gx (1985) which was published in part in several manuscripts.

**Table 3:** Light Microscopy (LM) studies compiled as part of the abundance compilation.

Reference	Method	Samples	Survey Period
Acri *et al*.^125^	LM	39	2015–2016
Assmy *et al*.^126^	LM	28	2004–2004
Dimiza *et al*.^101^	LM	9	2011–2011
Estrada *et al*.^127^	LM	229	1990–1990
Estrada *et al*.^76^	LM	400	2010–2011
Guerreiro *et al*.^74^	LM	150	2018–2018
Sal *et al*.^73^	LM	474	1992–2001
Valencia-Vila *et al*.^128^	LM	75	2000–2001
Estrada *et al*.^129^	LM	40	1985–1985
Grados *et al*.^130^	LM	9	1995–1995
Karentz^6^^†^	LM	1	2000–2000
Marshall *et al*.^131^	LM	32	1965–1965
Widdicombe *et al*.^79^	LM	482	1992–2008

^†^ dataset published as part of O’Brien *et al*.^6^.

Previous literature suggests that LM underestimates total coccolithophore abundances relative to SEM counts^70–72^. This is due to difficulty identifying lightly calcified taxonomic units with LM relative to SEM observations. To test if this holds for observations of the same taxonomic unit (instead of total coccolithophore abundance counts), we re-analysed data presented in Bollman *et al*.^70^ who thoroughly compared taxonomic-unit-specific coccolithophore abundance estimates using both SEM and LM measurements from the same water column-samples. However, rather than comparing total coccolithophore abundances, we compared taxonomic-unit-specific observations only. Furthermore, we grouped abundances of sub-species and varieties. We estimated the percentage difference between LM and SEM abundance estimates for each sample and then conducted Bayesian bootstrapping to find the mean percentage difference. We found that abundance estimates from LM differ by ≈ 19% (95% CI [1, 47%], Fig. 7). Thus, we did not consider the sampling method when converting our compilation of global abundances into POC and PIC stocks. Abundances were converted into POC and PIC by multiplying cellular carbon content distributions for each taxonomic unit by abundance values at each sample location.

The dataset includes data from 11 Atlantic Meridional Transect (AMT) cruises that occurred between 1995-2018. This data comes from three different publications: AMT 1-6^73^, AMT 12, 14, 15 and 17^61^, and AMT 28^74^. Additionally, we included all the sampling efforts conducted by Okada, Honjo, and Mclntyre in the Pacific and Atlantic Oceans between 1968-1972^41,75^, which were previously unavailable to the community. Other noteworthy datasets are data from Snellius-II Expedition^63^, the Malaspina-2010 expedition^76^, and a series of expeditions in the North Atlantic from 1987 to 1995^77,78^. The dataset also contains data from several time series, such as the English Channel ‘L4’ time series^79^, the Bermuda ‘BATS’ time series (1991-1994)^80^, the Hawaii ‘HOTS’ time series (1994-1996, 2004)^81,82^, and two a mesotrophic coastal ecosystem time series in the Adriatic Sea: ‘RV-001’ (2008-2009)^71^, and the ‘C1-LTER1’ (2011-2013)^72^.

#### Data grid and number of observations

The abundance compilation is provided in a non-gridded concatenated form and a gridded version with a 1-degree resolution with 5-m depth levels and a monthly temporal resolution. Gridding was conducted using Pandas in Python^67^. Mean values were utilised for grid cells with more than one measurement.

The concatenated dataset contains 6,166 samples for 139 taxonomic units and 42,975 abundance observations. The gridded dataset comprises 4,307 samples and 33,119 abundance observations.

#### Spatial-temporal bias

Within our dataset, most of the samples were collected within the top 50 m of the water column and are biased towards the northern hemisphere and Atlantic Ocean (Fig. 2). Nonetheless, the Southern Ocean is well represented in our compilation, covering every major Ocean basin. The fewest samples are observed in the winter and below 100 m.

## Data Records

The CASCADE dataset is available at Zenodo (zenodo.12794780)^25^. The repository contains five main folders: 1) “Concatenated literature”, which contains the merged datasets of size, PIC and POC and which were corrected for taxonomic unit synonyms; 2) “Resampled cellular datasets”, which contains the resampled datasets of size, PIC and POC in long format as well as a summary table; 3) “Gridded datasets”, which contains gridded datasets of abundance, PIC and POC; 4) “Classification”, which contains YAML files with synonyms, family-level classifications, and life cycle phase associations and definitions; 5) “Species list”, which contains spreadsheets of the “common” (>20 obs) and “rare” (<20 obs) species and their number of observations. Within the data archive a README file is provided describing the directory structure in detail.

## Technical Validation

### Species misspellings and synonyms

Taxonomic units were checked for synonyms and misspellings following taxonomy as reported on the NannoTax3 website^24^. During compilation, taxonomic units were checked to be in our database, and if flagged to be absent, they were added as synonyms or as new taxonomic units in the database.

### Spatial-temporal location

Latitude, longitude, date, and depth were checked to be of the correct datatype and within the expected range.

### Abundance observations

Cell concentrations were compared to the original publication and converted to cells per litre where appropriate.

### Cell size

Where multiple size measurements were available for a single taxonomic unit, we compared cell size estimates between studies and flagged any taxonomic units which showed greater than 5-fold disagreement between studies. Estimates for such taxonomic units were compared against the original literature, and dropped were appropriate. Dropped values included estimates of *Syracosphaera pulchra* HET and *Helicosphaera wallichii* HET from Villiot *et al*.^7^. Considerable disagreements in volume estimates were also observed for *Syracosphaera prolongata*; however, due to the unusual nature of the coccosphere morphology and the extensive range of size estimates reported in the literature, all estimates were kept in the compilation.

### POC and PIC regression

We checked the performance of our POC GLM and PIC GLM for Mean Average Error (MAE), Root Mean Squared Error (RMSE), and Cox and Snell pseudo-R-squared. Size, POC and PIC values were checked to be strictly positive and finite before fitting the GLMs.

### Supplementary information

Supplementary Information

## Data Availability

All code used to generate and validate the dataset is publicly available on GitHub (https://github.com/nanophyto/CASCADE)^83^. All pipelines were written in Python (3.11.4) and can be reproduced by running the Jupyter notebooks^84^ included in the repository. The Python package dependencies required to reproduce CASCADE, are provided in a YAML file, and can be installed to an Anaconda environment^85^ by following the instructions provided in the CASCADE GitHub README.
