# Supplementary material for: CASCADE: Dataset of extant coccolithophore size, carbon content and global distribution
Source: Sci Data. 2024 Aug 24;11:920. doi: 10.1038/s41597-024-03724-z (PMC11344785; doi:10.1038/s41597-024-03724-z)
Supplement: Supplementary file 1 — Supplementary Information [file 41597_2024_3724_MOESM1_ESM.pdf]

Supplementary Information

Species	Alternate phase	Reference
Acanthoica acanthifera HET	-	-
Acanthoica acanthos HET	-	-
Acanthoica quattrospina HET	*A. quattrospina* HOL	Cros et al., 2000[1]
		Cros et al., 2002[2]
		Supraha et al., 2014[3]
		Triantaphyllou et al., 2015[4]
Acanthoica quattrospina HOL	*A. quattrospina* HET	Cros et al., 2000[1]
		Cros et al., 2002[2]
		Supraha et al., 2014[3]
		Triantaphyllou et al., 2015[4]
Algirosphaera robusta HET	-	-
Alisphaera capulata HET	-	-
Alisphaera extenta HET	-	-
Alisphaera gaudii HET	-	-
Alisphaera gaudii POL	-	-
Alisphaera pinnigera HET	-	-
Alisphaera unicornis HET	*A. unicornis* POL	Supraha et al., 2018[5]
Alisphaera unicornis POL	*A. unicornis* HET	Supraha et al., 2018[5]
Calcidiscus leptoporus HET	*C. leptoporus* HOL	Kleijne et al., 1991[6]
		Cortes et al., 2000[7]
		Renaud et al., 2001[8]
		Houdan et al., 2004[9]
		Geisen et al., 2002[10]
Calcidiscus leptoporus HOL	*C. leptoporus* HET	Kleijne et al., 1991[6]
		Cortes et al., 2000[7]
		Renaud et al., 2001[8]
		Houdan et al., 2004[9]
		Geisen et al., 2002[10]
Calciopappus caudatus HET	-	-
Calciopappus rigidus HET	-	-
Calciosolenia brasiliensis HET	-	-
Calciosolenia murrayi HET	-	-
Calicasphaera blokii HOL	-	-
Calicasphaera concava HOL	-	-
Calicasphaera diconstricta HOL	-	-
Calyptrolithina divergens HOL	*S. halldalii* HET	Triantaphyllou et al., 2004[11]
Calyptrolithina multipora HOL	-	-
Calyptrosphaera heimdaliae HOL	*C. aculeata* HET	Triantaphyllou et al., 2015[4]
Calyptrosphaera sphaeroidea HOL	*C. sphaeroidea* HET	Noël et al., 2004[12]
Ceratolithus cristatus CER	*C. cristatus* HET	Archontikis et al., 2020[13]
		Alcober et al., 1997[14]
		Young et al., 1998[15]
		Cros et al., 2000[1]
		Sprengel et al., 2000[16]
Ceratolithus cristatus HET	*C. cristatus* CER	Archontikis et al., 2020[13]
		Alcober et al., 1997[14]

		Young et al., 1998[15]
		Cros et al., 2000[1]
Coccolithus pelagicus HET	*C. pelagicus* HOL	Geissen et al., 2002[17]
		Parke et al., 1960[18]
Coccolithus pelagicus HOL	*C. pelagicus* HET	Geissen et al., 2002[17]
		Parke et al., 1960[18]
Corisphaera gracilis HOL	*R. xiphos* HET	Supraha et al., 2018[5]
		NannoTax3[19]
Corisphaera tyrrheniensis HOL	-	-
Cyrtosphaera aculeata HET	-	-
Discosphaera tubifera HET	-	-
Emiliania huxleyi HET	-	-
Florisphaera profunda NANO	-	-
Flosculosphaera calceolariopsis HOL	-	-
Formonsella pyramidosa HET	-	-
Gephyrocapsa ericsonii HET	-	-
Gephyrocapsa muellerae HET	-	-
Gephyrocapsa oceanica HET	-	-
Gephyrocapsa ornata HET	-	-
Gephyrocapsa parvula HET	-	-
Gladiolithus flabellatus HET	-	-
Gliscolithus amitakareniae HOL	-	-
Hayaster perplexus HET	-	-
Helicosphaera HOL *catilliferus*	*H. carteri* HET	Cros et al., 2000[1]
		Cros et al., 2002[2]
Helicosphaera HOL *confusus*	-	-
Helicosphaera HOL *ponticuliferus*	*H. wallichii* HET	Couapel et al., 2009[20]
Helicosphaera carteri HET	-	-
Helicosphaera hyalina HET	-	-
Helicosphaera pavimentum HET	-	-
Helicosphaera pavimentum HOL	*H. pavimentum* HET	-
Helicosphaera wallichii HET	-	-
Helladosphaera cornifera HOL	*S. nodosa* HET	Cros et al., 2000[1]
	S. noroitica HET	Young et al., 2002[21]
Helladosphaera pienaarii HOL	-	-
Helladosphaera vavilovii HOL	-	-
Holococcolithophora dentata HOL	-	-
Homozygosphaera spinosa HOL	-	-
Homozygosphaera triarcha HOL	-	-
Michaelsarsia adriaticus HET	-	-
Michaelsarsia elegans HET	-	-
Oolithotus antillarum HET	-	-
Oolithotus fragilis HET	-	-
Ophiaster formosus HET	-	-
Ophiaster hydroideus HET	-	-
Palusphaera vandelii HET	-	-
Pappomonas type 3 HET	-	-
Pappomonas type 5 HET	-	-
Papposphaera lepida HET	-	-
Picarola margalefii HET	-	-
Pontosphaera syracusana HET	-	-
Poritectolithus maximus HOL	-	-
Poritectolithus poritectus HOL	-	-
Rhabdosphaera clavigera HET	-	-
Rhabdosphaera xiphos HET	-	-
Scyphosphaera apsteinii HET	-	-

Sphaerocalyptra adenensis HOL	-	-
Sphaerocalyptra quadridentata HOL	*A. robusta* HET	Kamptner et al., 1940[22]
		Triantaphyllou et al., 2003[23]
	A. robusta HET	Dimiza et al., 2008[24]
		Cros et al., 2002[2]
Sphaerocalyptra youngii HOL	-	-
Syracolithus sp. type A HOL	-	-
Syracosphaera ampliora HET	-	-
Syracosphaera anthos HET	*S. anthos* HOL	Cros et al., 2000[1]
Syracosphaera anthos HOL	*S. anthos* HET	Cros et al., 2000[1]
Syracosphaera arethusae HET	*S. arethusae* HOL	Cros et al., 2000[1]
		Triantaphyllou et al., 2015[4]
		Supraha et al., 2016[25]
Syracosphaera arethusae HOL	*S. arethusae* HET	Cros et al., 2000[1]
		Triantaphyllou et al., 2015[4]
		Supraha et al., 2016[25]
Syracosphaera aurisinae HET	*S. aurisinae* HOL	Keuter et al., 2021[26]
Syracosphaera aurisinae HOL	*S. aurisinae* HET	Keuter et al., 2021[26]
Syracosphaera bannockii HET	*S. bannockii* HOL	Cros et al., 2000[1]
		Daniels et al., 2014[27]
Syracosphaera bannockii HOL	*S. bannockii* HET	Cros et al., 2000[1]
		Daniels et al., 2014[27]
Syracosphaera borealis HET	-	-
Syracosphaera corolla HET	-	-
Syracosphaera dilatata HET	-	-
Syracosphaera epigrosa HET	-	-
Syracosphaera exigua HET	-	-
Syracosphaera gaarderae HET	*S. gaarderae* HOL	Keuter et al., 2019[28]
Syracosphaera gaarderae HOL	*S. gaarderae* HET	Keuter et al., 2019[28]
Syracosphaera halldalli HET	-	-
Syracosphaera hastata HET	-	-
Syracosphaera histrica HET	*S. histrica* HOL	Cros et al., 2000[1]
		Triantaphyllou et al., 2014[29]
		Triantaphyllou et al., 2015[4]
Syracosphaera histrica HOL	*S. histrica* HET	Cros et al., 2000[1]
		Triantaphyllou et al., 2014[29]
		Triantaphyllou et al., 2015[4]
Syracosphaera isselii HOL	-	-
Syracosphaera lafourcadii HOL	-	-
Syracosphaera magnaghii HOL	-	-
Syracosphaera marginiporata HET	-	-
Syracosphaera mediterranea HET	HOL *gracillima* type	Cortes et al., 2002[30]
		Triantaphyllou et al., 2015[4]
	HOL *marisrubri* type	Keuter et al., 2021[26]
	HOL *wettsteinii* type	Kamptner et al., 1940[22]
		Cros et al., 2000[1]
S. mediterranea HOL *gracillima* type	*S. mediterranea* HET	Cortes et al., 2002[30]
		Triantaphyllou et al., 2015[4]
S. mediterranea HOL *marisrubri* type	*S. mediterranea* HET	Keuter et al., 2021[26]
S. mediterranea HOL *wettsteinii* type	*S. mediterranea* HET	Kamptner et al., 1940[22]
		Cros et al., 2000[1]
Syracosphaera molischii HET	*S. molischii* HOL	Cros et al., 2000[1]
		Triantaphyllou et al., 2016[31]
		Skejic et al., 2018[32]
Syracosphaera molischii HOL	*S. molischii* HET	Cros et al., 2000[1]
		Triantaphyllou et al., 2016[31]

		Skejic et al., 2018 [32]
Syracosphaera nana HOL	*S. nana* HET	Kleijne et al., 1991 [6]
		Cros et al., 2000 [1]
Syracosphaera neapolitana HET	-	-
Syracosphaera nodosa HET	-	-
Syracosphaera noroitica HET	-	-
Syracosphaera orbiculus HET	-	-
Syracosphaera ossa HET	-	-
Syracosphaera periperforata HOL	*S. periperforata* HET	Archontikis et al., 2020 [13]
Syracosphaera prolongata HET	-	-
Syracosphaera protrudens HET	-	-
Syracosphaera pulchra HET	HOL *galea* type	Keuter et al., 2021 [26]
	HOL *oblonga* type	Cros et al., 2000 [1]
		Geissen et al., 2002 [17]
	HOL *pirus* type	Lecal-Schlauder 1961
		Geissen et al., 2002 [17]
		Heimdal et al., 2000 [33]
S. pulchra HOL *galea* type	*S. pulchra* HET	Keuter et al., 2021 [26]
S. pulchra HOL *oblonga* type	*S. pulchra* HET	Cros et al., 2000 [1]
		Geissen et al., 2002 [17]
S. pulchra HOL *pirus* type	*S. pulchra* HET	Lecal-Schlauder 1961 [34]
		Geissen et al., 2002 [17]
		Heimdal et al., 2000 [33]
Syracosphaera reniformis HET	-	-
Syracosphaera rotula HET	-	-
Syracosphaera squamosa HET	-	-
Syracosphaera strigilis HET	*S. strigilis* HOL	Supraha et al., 2015 [35]
		Supraha et al., 2018 [5]
Syracosphaera strigilis HOL	*S. strigilis* HET	Supraha et al., 2015 [35]
		Supraha et al., 2018 [5]
Syracosphaera tumularis HET	-	-
Turrilithus latericioides HET	-	-
Umbellosphaera irregularis HET	-	-
Umbellosphaera tenuis HET	-	-
Umbilicosphaera anulus HET	-	-
Umbilicosphaera foliosa HET	-	-
Umbilicosphaera hulburtiana HET	-	-
Umbilicosphaera sibogae HET	-	-
Wigwamma antarctica HET	-	-
Zygosphaera amoena HOL	-	-
Zygosphaera marsilii HOL	-	-

Supplementary table 1: Life cycle associations used in this study.

References

[1] Cros, L., Kleijne, A., Zeltner, A., Billard, C. & Young, J. New examples of holococcolith–heterococcolith combination coccospheres and their implications for coccolithophorid biology. *Marine Micropaleontology* **39**, 1–34 (2000).

[2] Cros, L. & Fortuño, J. M. Atlas of northwestern mediterranean coccolithophores. *Scientia Marina* **66**, 1–182 (2002).

[3] Supraha, L., Ljubesic, Z., Mihanovic, H. & Henderiks, J. Observations on the life cycle and ecology of acanthoica quattrospina lohmann from a mediterranean estuary. In *Journal of Nannoplankton Research*, vol. 34, 49–56 (2014).

[4] Triantaphyllou, M. V. *et al.* Coccolithophore combination coccospheres from the ne mediterranean sea: new evidence and taxonomic revisions. *Micropaleontology* 457–472 (2015).

[5] Šupraha, L., Ljubešić, Z. & Henderiks, J. Combination coccospheres from the eastern adriatic coast: New, verified and possible life-cycle associations. *Marine Micropaleontology* **141**, 23–30 (2018).

[6] Kleijne, A. Holococcolithophorids from the indian ocean, red sea, mediterranean sea and north atlantic ocean. *Marine micropaleontology* **17**, 1–76 (1991).

[7] Cortés, M. Further evidence for the heterococcolith–holococcolith combination calcidiscus leptoporus–crystallolithus rigidus. *Marine Micropaleontology* **39**, 35–37 (2000).

[8] Renaud, S. & Klaas, C. Seasonal variations in the morphology of the coccolithophore calcidiscus leptoporus off bermuda (n. atlantic). *Journal of Plankton Research* **23**, 779–795 (2001).

[9] Houdan, A. *et al.* Holococcolithophore- heterococcolithophore (haptophyta) life cycles: Flow cytometric analysis of relative ploidy levels. *Systematics and Biodiversity* **1**, 453–465 (2004).

[10] Geisen, M. *et al.* Life-cycle associations involving pairs of holococcolithophorid species : intraspecific variation or cryptic speciation? *European Journal of Phycology* **36**, 531–550 (2002).

[11] Triantaphyllou, M. V., Dimiza, M. D. & Dermitzakis, M. D. Syracosphaera halldalii and calyptrolithina divergens var. tuberosa life-cycle association and relevant taxonomic remarks. *Micropaleontology* **50**, 121–126 (2004).

[12] Nöel, M.-H., Kawachi, M. & Inouye, I. Induced dimorphic life cycle of a coccolithophorid, calyptrosphaera sphaeroidea (prymnesiophyceae, haptophyta) 1. *Journal of Phycology* **40**, 112–129 (2004).

[13] Archontikis, O. & Young, J. R. Extant ceratolithus cristatus life-cycle observations and taxonomic simplification. *Journal of Nannoplankton Research* **38** (2020).

[14] Alcober, J. & Jordan, R. An interesting association between neosphaera coccolithomorpha and ceratolithus cristatus (haptophyta). *European Journal of Phycology* **32**, 91–93 (1997).

[15] Young, J. R. Neogene nannofossils. *Calcareous Nannofossil Biostratigraphy. British Micropalaeontology Society Publications Series, Cambridge: Kluwer Academic Publisher* 225–265 (1998).

[16] Sprengel, C. & Young, J. First direct documentation of associations of ceratolithus cristatus ceratoliths, hoop-coccoliths and neosphaera coccolithomorpha planoliths. *Marine Micropaleontology* **39**, 39–41 (2000).

[17] Geisen, M. *et al.* Life-cycle associations involving pairs of holococcolithophorid species: intraspecific variation or cryptic speciation? *European Journal of Phycology* **37**, 531–550 (2002).

[18] Parke, M. & Adams, I. The motile (crystallolithus hyalinus gaarder & markali) and non-motile phases in the life history of coccolithus pelagicus (wallich) schiller. *Journal of the Marine Biological Association of the United Kingdom* **39**, 263–274 (1960).

[19] Young, J. R., Bown, P., Howe, R. & Lees, J. Nannotax3 website. https://www.mikrotax.org/ (2024). Accessed: 2024-02-05.

[20] Couapel, M. J., Beaufort, L. & Young, J. R. A new helicosphaera-syracolithus combination coccosphere (haptophyta) from the western mediterranean sea 1. *Journal of phycology* **45**, 914–916 (2009).

[21] Young, J. R. & Geisen, M. Xenospheres-associations of coccoliths resembling coccospheres. *Journal of Nannoplankton Research* **24**, 27–35 (2002).

[22] Kamptner, E. Die coccolithineen der südwestküste von istrien. *Annalen des Naturhistorischen Museums in Wien* 54–149 (1940).

[23] Triantaphyllou, M. & Dimiza, M. Verification of the algirosphaera robusta–sphaerocalyptra quadridentata (coccolithophores) life-cycle association. *Journal of Micropalaeontology* **22**, 107–111 (2003).

[24] Dimiza, M. D., Triantaphyllou, M. V. & Dermitzakis, M. D. Seasonality and ecology of living coccolithophores in eastern mediterranean coastal environment (andros island, middle aegean sea). *Micropaleontology* 159–175 (2008).

[25] Šupraha, L., Ljubešić, Z., Mihanović, H. & Henderiks, J. Coccolithophore life-cycle dynamics in a coastal mediterranean ecosystem: Seasonality and species-specific patterns. *Journal of Plankton Research* **38**, 1178–1193 (2016).

[26] Keuter, S., Young, J. R., Koplovitz, G., Zingone, A. & Frada, M. J. Novel heterococcolithophores, holococcolithophores and life cycle combinations from the families syracosphaeraceae and papposphaeraceae and the genus florisphaera. *Journal of Micropalaeontology* **40**, 75–99 (2021).

[27] Daniels, C. J., Tyrrell, T., Poulton, A. J. & Young, J. R. A mixed life-cycle stage bloom of syracosphaera bannockii (borsetti and cati, 1976) cros et al. 2000 (bay of biscay, april 2010). *Journal of Nannoplankton Research* **34**, 31–35 (2014).

[28] Keuter, S., Young, J. R. & Frada, M. J. Life cycle association of the coccolithophore syracosphaera gaarderae comb. nov.(ex alveosphaera bimurata): Taxonomy, ecology and evolutionary implications. *Marine Micropaleontology* **148**, 58–64 (2019).

[29] Triantaphyllou, M. V., Dimiza, M. D. & Karatsolis, B. Adding proofs to syracosphaera histrica-calyptrolithophora pappilifera life-cycle association. *J. Nannoplankt. Res* **34**, 57–59 (2014).

[30] Cortes, M. Y. & Bollmann, J. A new combination coccosphere of the heterococcolith species coronosphaera mediterranea and the holococcolith species calyptrolithophora hasleana. *European Journal of Phycology* **37**, 145–146 (2002).

[31] Triantaphyllou, M. V. *et al.* Coccolithophore combination coccospheres from the ne mediterranean sea: new evidence and taxonomic revisions. *Micropaleontology* 457–472 (2015).

[32] Skejić, S. *et al.* Coccolithophore diversity in open waters of the middle adriatic sea in pre-and post-winter periods. *Marine Micropaleontology* **143**, 30–45 (2018).

[33] Heimdal, B. R. & Saugestad, A. H. Light microscope studies on coccolithophorids from the western mediterranean sea, with notes on combination cells of daktylethra pirus and syracosphaera pulchra. *Plant Biosystems-An International Journal Dealing with all Aspects of Plant Biology* **136**, 3–27 (2002).

[34] Lecal-Schlauder, J. Anomalies dans la composition des coques de flagelles calcaires. *Bulletin de la Societe d'Histoire Naturelle de l'Afrique du Nord* **52**, 63–66 (1961).

[35] Šupraha, L., Gerecht, A. C., Probert, I. & Henderiks, J. Eco-physiological adaptation shapes the response of calcifying algae to nutrient limitation. *Scientific Reports* **5**, 16499 (2015).
